# Chiral Porous Carbon Surfaces for Enantiospecific Synthesis

**DOI:** 10.3390/polym14142765

**Published:** 2022-07-06

**Authors:** Sapir Shekef Aloni, Molhm Nassir, Yitzhak Mastai

**Affiliations:** Department of Chemistry and Institute of Nanotechnology and Advanced Materials (BINA), Bar-Ilan University, Ramat-Gan 5290002, Israel; sap.shekef@gmail.com (S.S.A.); molhmnas@gmail.com (M.N.)

**Keywords:** chirality, chiral surface, ionic liquids, porous carbon, chiral catalysis

## Abstract

Chiral surfaces, developed in the last decade, serve as media for enantioselective chemical reactions. Until today, they have been based mostly on developments in silica templating, and are made mainly from imprints of silicate materials developed a long time ago. Here, a chiral porous activated carbon surface was developed based on a chiral ionic liquid, and the surface chemistry and pore structure were studied to lay a new course of action in the field. The enantioselectivities of surfaces are examined by using variety of methods such as circular dichroism, linear sweep voltammetry and catalysis. These techniques revealed a 28.1% preference for the D enantiomer of the amino acid proline, and linear sweep voltammetry confirmed chirality recognition by another probe. An aldol surface chiral catalytic reaction was devised and allowed to determine the root of the enantiomeric excess. These results affirm the path toward a new type of chiral surface.

## 1. Introduction

Since Louis Pasteur first realized chemical chirality in 1848, its importance and study progressed greatly. Enantiomers exhibit identical chemical and physical properties, yet their interactions with biological compounds, e.g., proteins, polysaccharides or DNA, are quite different. Due to this important aspect, chiral molecules were studied extensively over the years, and most of the effort was focused on chiral molecules in liquid solutions or solid crystals. However, in the last two decades, there has been a significant increase in the study of chiral surfaces based on both achiral and chiral materials.

Chiral surfaces have been among the most attractive topics in the chemical community [1,2,3,4,5,6,7,8,9] and play an important role in nanotechnology—for example, in chiral nanoscale systems, chiral surfaces [10,11,12,13,14,15,16,17,18,19], enantioselective synthesis, chiral separation, and chiral electrochemistry. In recent years, chiral carbon particles were made by carbonization of a chiral ionic liquid (CIL) [20,21,22]. Porous carbonaceous materials are very good adsorbents and have unique abilities, since they are low cost, light-weight, and constructed from natural raw materials. There are several glass forms of silica materials which fulfill this description, while crystalline forms of silica, such as zeolites, tend to have smaller pores in the microporous size range [23]. The first mesoporous hydrothermal carbons were produced by performing hydrothermal carbonization in the presence of nanostructured silica templates. Thus, it was found that it is important to match the polarity of the template surface with the one of the carbon precursors. For mesoporous templates, mesoporous carbon shells are obtained after removal of the template [24]. Carbon is in many ways the element of choice by nature. Its availability and chemical complexity make it a desired material in many applications. The increase demand for sustainable yet versatile and cost efficient “green” manufacturing brings the high-tech material industry to explore advantages in production manufacturing, and elements of choice more adequate to modern demands [25]. Moreover, chiral carbon can be utilized as a chiral adsorbent, and thus may be utilized e.g., in chiral separations by classic chromatographic methods or in asymmetric (electro)catalysis or chiral sensing.

Here, we reveal the innovative application of such particles for the preparation of stable chiral surfaces. Chiral porous carbon was prepared on various nickel surfaces such as foil and foams, and the carbonization process was performed on the Ni surfaces. Chemical and chiral functionalization of the surface of porous carbon materials is very challenging. Therefore, low-temperature formation of chiral surfaces by carbonization of CILs with chiral information could lead to a research breakthrough in the area of chiral surfaces. Such surfaces were prepared, and their chiral recognition was investigated by isothermal titration calorimetry, circular dichroism (CD) and electrochemical methods.

## 2. Materials and Methods

**Chemicals**. The chemicals for the synthesis were purchased from Aldrich-Sigma Germany: L-proline (>99%), D-proline (>98%), iodomethane (>99.8%), potassium hydrogen carbonate, L-tartaric acid (>98%) and, D-tartaric acid (>98%).

**Scheme 1:**
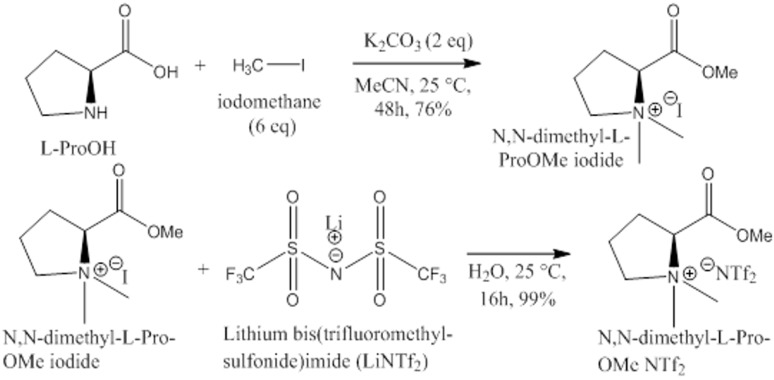
Complete schematic route for the synthesis of chiral ionic liquids (CILs).

**Synthesis of CILs.** The complete route for the synthesis of chiral ionic liquids is shown in Scheme 1. L- or D-proline (5.00 g, 43.47 mmol) was placed in a 500 mL round bottom flask, 120 mL of acetonitrile was added, and the mixture was stirred for 10 min; potassium hydrogen carbonate (12 g, 86.95 mmol, 2 eq) was added and stirred for 10 min, followed by the addition, of iodomethane (37.04 g, 260.86 mmol, 6 eq) and the suspension was stirred. After 48 h, the mixture was filtrated (to remove salts) and evaporated. The crude was stirred with chloroform (75 mL), and the remaining non-dissolved solid was removed to obtain N,N-dimethyl-L-proline methyl ester iodide. Ion exchange of the iodide for bis(trifluoromethyl-sulfonide) imide was performed by the following method:

Water (11.38 mL) was added to N,N-dimethyl-L-ProOMe iodide (5.69 g, 19.96 mmol), and the mixture was stirred. LiNTf_2_ (8.59 g, 29.9 mmol, 1.5 eq) was added, and the mixture was stirred to give a clear solution. The clear solution was stirred overnight at room temperature, after which the product was precipitated as a liquid (new bottom layer). Dichloromethane (17 mL) was added to the mixture, and after stirring, the upper aqueous layer was removed. The organic layer was washed with water (4 × 3 mL) and dried over magnesium sulfate. The mixture was filtered (by gravity) and evaporated to N,N-dimethyl-L-ProOMe NTf_2_ as an oil. The purity of the proline-based CILs product, which we term L- and D-CIL(Pro), was confirmed by 1H and 13C NMR spectroscopy and mass spectrometry (Scheme 1).

**Scheme 2:**
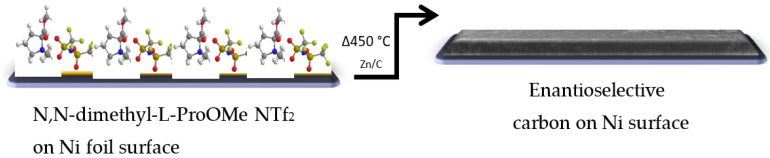
Formation of a chiral surface by carbonization of (L/D)-CIL(Pro).

**Carbonization process**. A CIL based on the specific amino acid proline was mixed and carbonized with an identical part of a eutectic mixture of NaCl and ZnCl_2_ salts (1:3 molar ratio). After the mixing, 1 g was weighed and spread on top of Ni foil (1 cm^2^). The coated foil was placed in a closed chamber furnace with N_2_ flow at 25 °C for 1 h, and the furnace was heated to 450 °C at a rate of 2.5 °C/min, maintained for 1 h, followed by gradual cooling to room temperature. The solid carbonization products were washed exclusively with water to extract remaining salts, resulting in a carbonized CIL material (Scheme 2) [26].

**Characterization.** The morphology of the CIL was studied with FEI Quanta FEG 250 scanning electron microscope (SEM) combined with EDAX and a Jeol-1400 (LaB6, 120 kV). The test for the presence of carbon in the CILs on the Ni foil surface was by X-ray diffraction (XRD) using a Rigaku Smart Lab 3 kW Advance diffractometer equipped with Cu Kα (λ = 1.5418 Å) applying 2θ between 3–80° and step size of 0.02° counting time of at 1 s per step. The Raman spectrum was recorded using a Witec (Focus Innovations) Raman microscope operating with an objective (Nikon 10×/0.25, ∞/-WD 6.1) at an excitation wavelength of 532 nm with an intensity of 3.5 mW to confirm the presence of carbon. Absorbance measurements were performed using a Chirascan circular dichroism (CD) spectrometer (Applied Photophysics, Leatherhead, UK) at a bandwidth of 3 nm, from 340 to 190 nm, with step size and duration of 1 nm and 3 sec, respectively.

## 3. Results and Discussion

**Figure 1:**
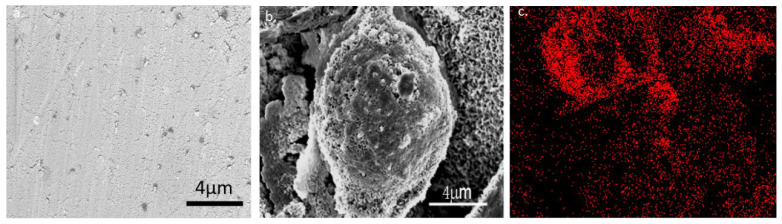
High-resolution scanning electron microscopy images of L-CIL(Pro)-C on nickel foil obtained by HR-SEM and EDAX. (**a**) pure Ni foil surface (**b**) carbonized Ni foil (**c**) EDAX mapping of carbon on the Ni surface of carbonized Ni foil.

A chiral ionic liquid (CIL) of amino acid proline was synthesized with two different counter ions: tetrafluoroborate (BF_4_) and bis(trifluoromethane) sulfonimide (NTf_2_) and carbonated onto the surface of nickel foil. However, as the ionic liquid of proline with BF_4_ had very poor adhesion to the Ni surface, and the carbonized material washed off the surface when we started cleaning the surface from the by-products of the carbonization. We decided to focus on ionic liquid of proline with NTf_2_. For all experiments we used L- or D-CIL(Pro)-C ionic liquid on Ni foil, namely L/D-CIL(Pro)-C//Ni foil. We started with some general characterization of the material as follows, to verify that we obtained a carbon material. The morphology of the carbon surfaces of L/D-CIL(Pro)-C//Ni foil was explored by HR-SEM and EDAX. As shown in Figure 1, the surface of L-CIL(Pro)-C//Ni foil reveals a spherical shape with particles having an average diameter of ca. 10 μm. Moreover, the Ni foil surfaces are homogeneously coated with a layer of carbonaceous material with porous structure and high surface roughness (Figure 1b). This is compared to a sample of pure Ni foil surface (Figure 1a). The presence of carbon at the surface of the particles is proved by energy-dispersive X-ray spectroscopy (EDX) elemental mapping (Figure 1c), which shows an almost even distribution of carbon on the Ni foil surface.

**Figure 2:**
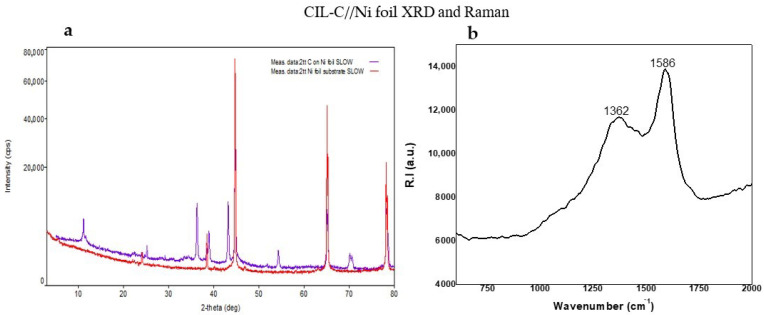
(**a**) Two-theta scans of L-CIL(Pro)-C on Ni foil (purple) and Ni foil substrate (red) with a grazing-incidence angle of 3–80°. (**b**) Raman spectrum of CIL(Pro)-C on Ni surface.

Next, we used X-ray diffraction (XRD) patterns to explore the structure of the carbonaceous CIL(Pro)-C layers formed on the Ni foil surfaces. Figure 2a displays the pattern of L-CIL(Pro)-C as an amorphous carbon structure such as C Carbon, C graphite [27,28] or theoretical graphite [29], as reported in the literature. The peaks at 2θ = 44.50 and 76.41 shown in Figure 2a (red line) correspond well with the two most intense peaks, (1,1,1) and (2,2,0), respectively, of face-centered cubic metallic Ni surface [29,30,31].

The so-called disordered (D) and graphite (G)-like bands in Raman spectroscopy were employed to analyze the sp^2^-based carbon structures on our samples (Figure 2b). The D band near 1350 cm^−1^ is caused by faults and disorder in the breathing modes of six-fold sp^2^-hybridized carbon rings, while the G band near 1590 cm^−1^ is caused by bond stretching of sp2-hybridized carbon in rings or chains. The amount of six-membered sp^2^ carbon rings is proportional to the peak height ratio of the D- and G-bands (ID/IG), which is often used to measure the level of carbon ordering in porous carbons [32].

To explore the thermal stability of the carbon surfaces we decided to perform thermogravimetric analysis (TGA) measurements that have shown thermal stability as reported in our previous articles. In brief, the carbon surfaces on Ni show weight loss of about 10 percent in the temperature range of 100 to 400 °C due to carbonization, and then the samples are stable up to 800 °C.

In conclusion, we have demonstrated a new path to synthesize porous carbon surfaces on Ni surfaces. Our synthesis is based on the carbonization process of chiral ionic liquids combined with a salt melt process [21,23]. It should be noted that in this article we presented the results of CIL of proline. However, we performed a synthesis of other chiral ionic liquid with tyrosine, phenylalanine and leucine, and similar results on the porous carbon surfaces were also obtained.

In the next step, we started to study the chiral recognition capability of our carbon based surfaces. Examination of enantioselectivity of CIL surfaces is of great interest for several applications, such as chiral separation and enantioselective adsorption [33]. Several approaches were developed for chiral recognition of CILs including chiral recognition of enantiomers, chiral recognition agents, and organocatalysts [34,35]. However, existing methods for measuring chiral recognition of CILs on surfaces are somewhat limited; therefore, proving the enantioselectivity of the CIL surfaces is a challenging task.

**Figure 3:**
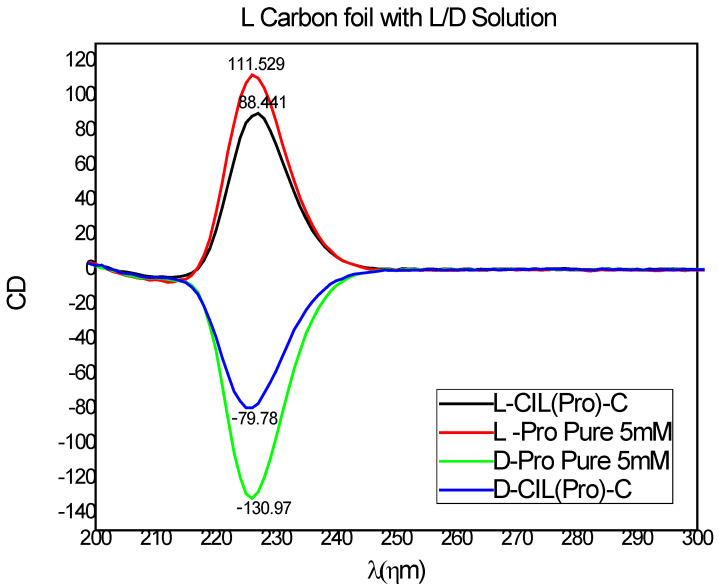
CD spectra of L-Pro probe (red) and D-Pro probe (green) of L- CIL(Pro)-C//Ni foil (black), and D- CIL(Pro)-C//Ni foil (blue).

We proceeded to study the chiral recognition of our carbon-based surfaces. An effort to investigate the enantioselectivity of our surface started with circular dichroism (CD) spectroscopy to prove the preferential adsorption of D- and L-amino acids on the CIL(Pro)-C//Ni foil surfaces. We chose to test the adsorption of D- and L-proline solutions as probes over the CIL(Pro)-C//Ni foil surfaces to demonstrate chiral recognition by the carbon surfaces. CD spectroscopy was used to detect differences in absorption of L- and D-proline (5 mM aqueous solutions) adsorption with and without A 1 × 1 cm^2^ surface of CIL(Pro)-C//Ni foil, and the CD signals obtained after adsorption equilibrium (after ~24 h) were measured. The enantiospecificity calculations and CD ellipticity of the pure L-and D-Pro solutions and adsorbed solutions are shown in Figure 3. Calculations of the CD signals obtained by adsorption of L- and D-Pro revealed significant differences between the two enantiomers, demonstrating stereoselective adsorption. The change in CD signal of the L enantiomer was ca. 23.00 mDeg, calculated as a change of 21.70%, while the CD signal of the D enantiomer was ca. 39.09 mDeg, calculated as a change of 51.19%. Overall, the adsorption experiments proved an enantioselectivity of 28.1% in favor of the D enantiomer. This value is relatively high for chiral synthesized materials and is sufficient for efficient enantiomeric separation on the chiral.

**Figure 4:**
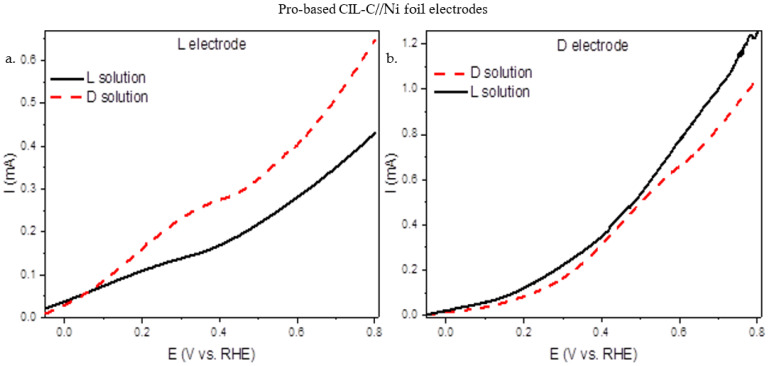
LSV (50 mV s^−1^) of Pro-based CIL-C electrodes: (**a**) L-CIL(Pro)-C//Ni foil electrode and (**b**) D- CIL(Pro)-C//Ni foil electrode with R,R/S,S-TA. LSV measurements were taken at room temperature in 5 mM R,R- or S,S-TA (0.1 M Na_2_SO4 electrolyte solution).

Electrochemical methods allow examination of many fundamental aspects of surfaces and make it possible to explore asymmetric reactions at solid surfaces in a very detailed manner. Important insights into the nature of the surface activity may be obtained by various electrochemical techniques. Linear sweep voltammetry (LSV [36,37]) is particularly sensitive to changes in the local charged state of different surface regions (terraces, steps, and kinks [38,39,40,41,42]), and hence allows us to identify chiral properties of these surface sites. The enantioselective properties of the carbon surface on Ni were tested by LSV. L- and D-CIL(Pro)-C//Ni foil were tested in an electrolyte solution with S,S or R,R tartaric acid (TA) as a chiral probe molecule. As a working electrode, a three-electrode cell was built using 1 cm^2^ of porous carbon. S,S- and R,R-TA (5 mM) chiral solutions were used for LSV experiments, using Na_2_SO_4_ (0.1 M) as a supporting electrolyte, and the electrochemical response was recorded between −0.1 V and 0.8 V vs. RHE. On carbon electrodes, electrochemical oxidation of TA occurs at about 0.6–0.7 V vs. RHE, according to the literature. [21,22,23,24,25,26,27]. Figure 4a shows the LSV curves obtained on a L-CIL(Pro)-C//Ni foil electrode with 5 mM R,R- and S,S-TA solution, while Figure 4b shows the same for a D-CIL(Pro)-C//Ni foil electrode. From the measurements (Figure 4), we can see a difference in the currents of the two enantiomeric carbon electrodes in response to the TA enantiomer. The L electrode exhibits a higher current towards R,R-TA oxidation, the opposite enantiomer, while for the corresponding enantiomer, the S,S-TA, the currents are lower (Figure 4a). The same trend is observed for the D electrode, where higher currents were measured for the D electrode with the S,S-TA (Figure 4b). It should be emphasized that the LSV measurements demonstrate chiral recognition that is coordinated with the chirality of the porous carbon in the CD. The electrochemical studies verify the chiral nature and enantioselective properties of the carbon electrodes.

**Scheme 3:**
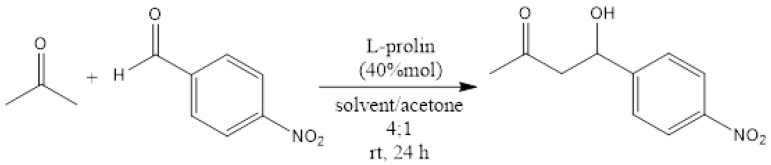
Aldol reaction to obtain 4-hydroxy-4-nitrophenylbutan-2-one (PNHB).

To back up these findings and show that the CIL(Pro)-C/Ni foil surface is enantioselective, we used it as an enantioselective catalyst for the well-known aldol stereoselective reaction. The asymmetric aldol reaction is among the most important carbon carbon bond formations in organic chemistry [24,43,44]. Amino acids and especially proline are very effective asymmetric catalysts for the direct aldol reaction. We performed several aldol reactions to examine the efficiency of CIL(Pro)-C//Ni foil surface material compared to the reaction under standard catalysis by pure proline in solution. An effective synthesis with a similar yield to the control synthesis was performed as follows: nitrobenzaldehyde (0.5 g, 3.3 mmol) and 0.2 mL of dry acetone were dissolved in DMF (1 mL), followed by the addition of L-Pro (4.6 mg, 0.04 mmol). The reaction mixture was stirred for 24 h at room temperature and monitored by TLC (98:02- hexane: ethyl acetate). The residue was extracted with ethyl acetate three times (20 mL), and the organic phase was dried with Mg_2_SO_4_ and concentrated under vacuum to give the product 4-hydroxy_-_4_-_nitrophenylbutan_-_2_-_one (PNHB) with 66% yield (Scheme 3). The product was measured by NMR and the results were as follows: 1H NMR (D_2_O, 400 MHz): δ = 8.12 (dd, J = 8.6, 1.8 Hz, 2H), 7.47 (d, J = 8.5 Hz, 2H), 5.19 (t, J = 6.1 Hz, 1H), 2.78 (d, J = 6.7 Hz, 2H), 2.17 (s, 3H) ppm; 13C NMR (D_2_O, 100.61 MHz): δ = 208, 150, 147, 126, 123, 68, 51, 30 ppm; experimental optical rotation in chloroform: ${\left[\propto \right]}_{598\;\mathrm{nm}}^{25{}^{\circ}}$ = 61.6. In our experiment, we changed the known L-Pro to L-CIL(Pro)-C//Ni foil surface and used the same reaction route to give the same enantioselective response reported in the control synthesis to receive PNHB. A set of small-scale experiments was used with constriction of 0.11 M instead of 0.51 M and 40 mol% of L-CIL(Pro)-C//Ni foil, and the optical rotation was ${\left[\propto \right]}_{598\;\mathrm{nm}}^{25{}^{\circ}}$ = 13.28°. The calculated enantiomeric excess was 21.55%. Another series of experiments focused on alternative solvents—DMSO and acetone—and evaluated whether the yield could be increased. We found that the ideal solvent for reactions with L-Pro and L-CIL(Pro)-C//Ni foil surface is THF, giving an average product yield of 60% for both reactions (Table 1).

## 4. Conclusions

In conclusion, for the first part of our work we describe the development of an innovative type of chiral porous carbon surface based on a unique carbonization process of chiral ionic liquids (CIL) precursors based on amino acids such as proline, tyrosine or phenylalanine. We demonstrated the chiral nature of these porous carbons by employing unique analytical techniques. We believe that the approach presented in this work is highly significant for the development of a new type of chiral porous materials for enantioselective chemistry. In addition, it demonstrates significant progress in the understanding of the structure and nature of chiral porous materials and nanosurfaces. The chiral nature of these porous carbon surfaces was identified using various measurements. For example, CD measurements showed that D-CIL(Pro)-C on Ni foil—D-CIL(Pro)-C//Ni—has an enantioselective preference over L-CIL(Pro)-C//Ni. Moreover, LSV measurements of L/D-CIL(Pro)-C//Ni foil electrode with S,S or R,R tartaric acid (TA) showed an enantioselective preference. The L electrode exhibits a higher current towards R,R-TA oxidation. The same trend was found for the D electrode, where higher currents were measured with S,S-TA.

The enantioselective surfaces were used in the aldol reaction instead of L/D-proline. The reaction with L-CIL(Pro)-C//Ni as a catalyst had effectively the same yield as L/D-Pro, opening up a new alternative for enantioselective catalyst materials for chiral catalytic reactions. We believe that this is only the beginning of a new era for chiral surface materials for enantioselective applications. Our pioneering study on chiral behavior will hopefully enlighten this field. This work gives a very preliminary example for the synthesis of chiral carbon surfaces, and we hope that in the future it will be possible to refine and develop this method using other metal surfaces and many other chiral ionic liquids. For many years, scientists have been investing efforts in the development of new methods for the synthesis of chiral surfaces and for chirality in the solid state. We believe that the method we propose in this article will expand the range of methods for synthesis of chiral surfaces, and will be developed and applied by many others.

## Figures and Tables

**Table 1:** Yield of PNHB in aldol reaction in different solvents.

No	Solvent	Yield (%)
1	DMSO	N.O ^1^
2	THF	60
3	DMF	16 ^2^

^1^ N.O not observed, main product was 4-hydroxypenta-2-one; ^2^ main product was 2-aldoxane.

## Data Availability

Not applicable.
